# Robot-Assisted Distal Gastrectomy with Preservation of a Congenital Portosystemic Shunt for Gastric Neuroendocrine Tumor: A Case Report

**DOI:** 10.70352/scrj.cr.26-0289

**Published:** 2026-07-11

**Authors:** Tsuyoshi Saito, Koshiro Harata, Rena Kunimatsu, Kaita Suzuki, Nobuaki Kagohashi, Ryosuke Niwamoto, Motoki Futamata, Shuji Koide, Takeyasu Katada, Futoshi Teranishi, Shuji Takiguchi

**Affiliations:** 1 Department of Gastroenterological Surgery, Toyokawa City Hospital, Toyokawa, Aichi, Japan; 2 Department of Gastroenterological Surgery, Nagoya City University Graduate School of Medical Sciences, Nagoya, Aichi, Japan

**Keywords:** congenital portosystemic shunt, gastrectomy, neuroendocrine tumor

## Abstract

**INTRODUCTION:**

Gastric neuroendocrine tumors (gNETs) are rare neoplasms; their coexistence with a congenital portosystemic shunt (CPSS) presents unique surgical challenges caused by the risks of intraoperative hemorrhage and postoperative hepatic dysfunction secondary to altered portal hemodynamics. To the best of our knowledge, no reports have described robot-assisted gastrectomy preserving a CPSS during gNET resection.

**CASE PRESENTATION:**

A 54-year-old man presented with gastrointestinal hemorrhage and was diagnosed with a gNET (G1). Contrast-enhanced CT revealed a CPSS connecting the left gastric vein (LGV) and left renal vein (LRV) adjacent to the stomach. Given the absence of hepatic dysfunction and the potential risk of portal hypertension after shunt interruption, preservation of the anomalous vessel was planned. Robot-assisted distal gastrectomy with D2 lymphadenectomy was performed. The robotic platform provided stable 3D visualization and facilitated precise dissection around the anomalous vessel, thereby enabling complete tumor resection with adequate margins while preserving the shunt. Histopathological examination confirmed a G1 neuroendocrine tumor, without lymph node metastasis. The postoperative course was uneventful, with no hepatic or oncological complications.

**CONCLUSIONS:**

Robot-assisted gastrectomy may facilitate safe and precise resection in selected patients with gNETs concurrent with CPSSs.

## Abbreviations

ALT
: alanine aminotransferase
AST
: aspartate transaminase
CHA
: common hepatic artery
CPSS
: congenital portosystemic shunt
gNET
: gastric neuroendocrine tumor
LGA
: left gastric artery
LGV
: left gastric vein
LRV
: left renal vein
PT-INR
: prothrombin time–international normalized ratio
PV
: portal vein
SpA
: splenic artery

## INTRODUCTION

gNETs are uncommon neoplasms, accounting for approximately 0.6%–2% of all gastric malignancies.^1)^ According to the 2019 World Health Organization classification (WHO), neuroendocrine tumors are graded as G1, G2, or G3 based on the mitotic count and Ki-67 proliferation index.^2)^

A CPSS is a rare vascular anomaly characterized by the diversion of portal venous blood into the systemic circulation without passing through the liver. Although some patients remain asymptomatic, a CPSS may lead to hyperammonemia, hepatic dysfunction, and coagulopathy. During abdominal surgery, this anomalous vascular anatomy may increase the risk of intraoperative bleeding and postoperative hepatic complications.

The coexistence of a gNET and a CPSS is extremely rare. To our knowledge, we could not identify any previous report describing robot-assisted gastrectomy with preservation of a CPSS for gNET. Herein, we present this case to emphasize the importance of detailed preoperative evaluation and individualized surgical planning for such complex anatomical anomalies.

## CASE PRESENTATION

A 54-year-old man with a history of hypertension and type 2 diabetes presented with melena. Blood tests performed by his primary care physician revealed progressive anemia, and he was referred to our hospital for further evaluation.

Upper gastrointestinal endoscopy revealed a raised ulcerative lesion on the anterior wall of the mid-body of the stomach (**Fig. 1**). Biopsy demonstrated atypical cells with round nuclei arranged in rosette-like clusters within the lamina propria. Immunohistochemistry was positive for chromogranin A, synaptophysin, and CD56, confirming the diagnosis of a gNET. The Ki-67 labeling index was <1%, consistent with a G1 gNET according to the 2019 WHO classification.

**Fig. 1:**
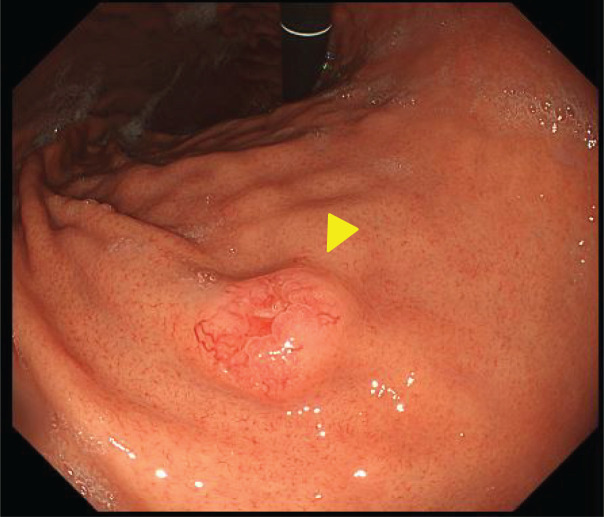
Upper gastrointestinal endoscopic findings. Upper gastrointestinal endoscopy reveals an elevated erosive lesion on the anterior wall of the gastric mid-body (arrowhead).

Endoscopic ultrasonography identified a 13-mm lesion confined to the second layer of the anterior wall of the mid-body of the stomach (**Fig. 2**). Contrast-enhanced abdominal CT did not clearly identify the tumor but revealed a markedly dilated LGV draining into the LRV, forming a CPSS adjacent to the stomach (**Fig. 3**). The shunt vessels were uniform in diameter, without tortuosity, collateral veins, or varices, consistent with a congenital origin. No splenomegaly or additional shunts were observed.

**Fig. 2:**
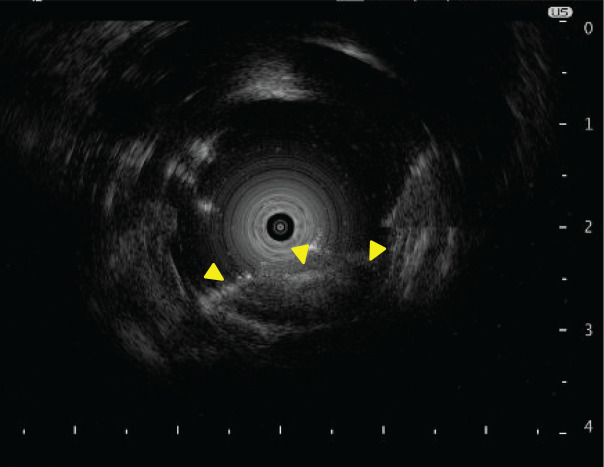
Endoscopic ultrasonography findings. Endoscopic ultrasonography reveals a 13-mm lesion confined to the second layer of the anterior wall of the gastric mid-body (arrowheads).

**Fig. 3:**
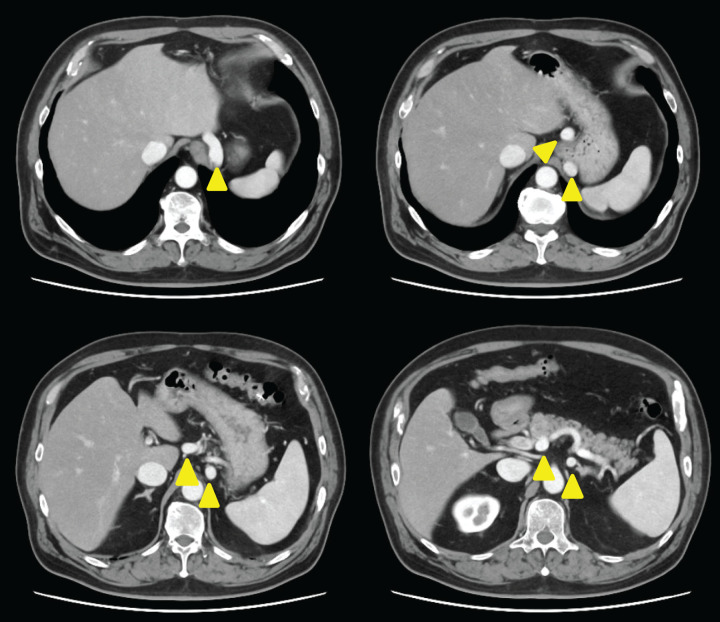
Contrast-enhanced CT findings. A markedly dilated LGV arising from the PV courses along the anterior wall of the gastric cardia and drains dorsally into the LRV (arrowheads). However, the gastric tumor is not clearly identifiable on CT. LGV, left gastric vein; LRV, left renal vein; PV, portal vein

Laboratory data were as follows: AST 19 U/L, ALT 19 U/L, total bilirubin 0.4 mg/dL, PT-INR 0.99, serum ammonia 45.3 μg/dL, and serum gastrin 26.1 pg/mL, all within normal limits. Based on these findings, we planned to perform a robot-assisted distal gastrectomy while preserving the shunt vessel. Preoperative 3D CTA was performed to evaluate the vascular anatomy (**Fig. 4**).

**Fig. 4:**
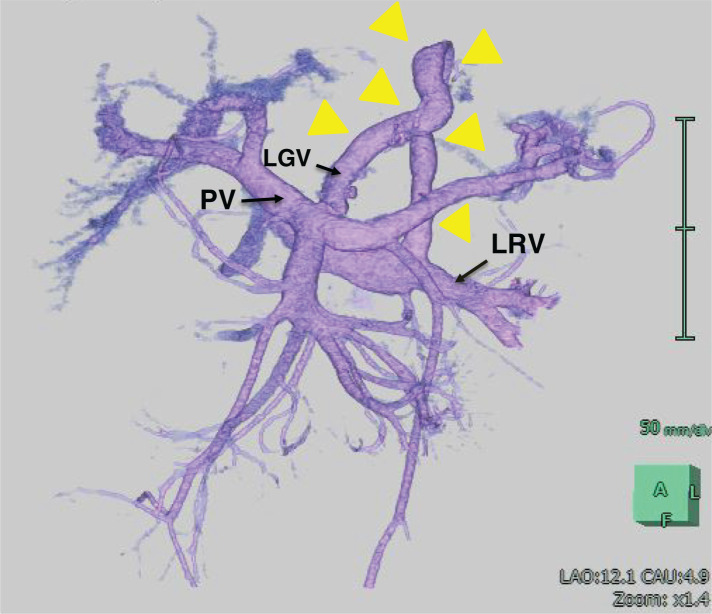
3D CTA of the venous system. 3D CTA clearly demonstrates a CPSS, in which a markedly dilated LGV with a uniformly enlarged diameter originates from the main PV trunk, courses dorsally to form a cranial arch, and drains into the LRV without evidence of varices or collateral vessel formation (arrowhead). Continuity between the PV trunk and the intrahepatic portal branches is preserved. CAU, caudal; CPSS, congenital portosystemic shunt; LAO, left anterior oblique; LGV, left gastric vein; LRV, left renal vein; PV, portal vein

After pneumoperitoneum was established, the abdominal cavity was inspected using a da Vinci camera inserted through the umbilical port. The liver exhibited a normal color and sharp margins, and no macroscopic findings suggestive of liver cirrhosis were observed. The remaining ports were placed, the da Vinci system was docked, and robot-assisted surgery was initiated.

The omental bursa was opened, and lymphadenectomy along the greater curvature was performed, followed by duodenal transection. The procedure was then advanced to the suprapancreatic lymph node dissection. After completing the lymphadenectomy around the CHA, a markedly dilated LGV branching from the PV was identified.

After identifying the LGA running along the left side of the LGV, which constituted the main trunk of the CPSS (**Fig. 5A**), the artery was clipped and divided, and the proximal dissection line was determined. Lymphadenectomy was then initiated along the preserved LGV while separating the lymphatic tissue from the vascular wall to facilitate en bloc dissection (**Fig. 5B**).

**Fig. 5:**
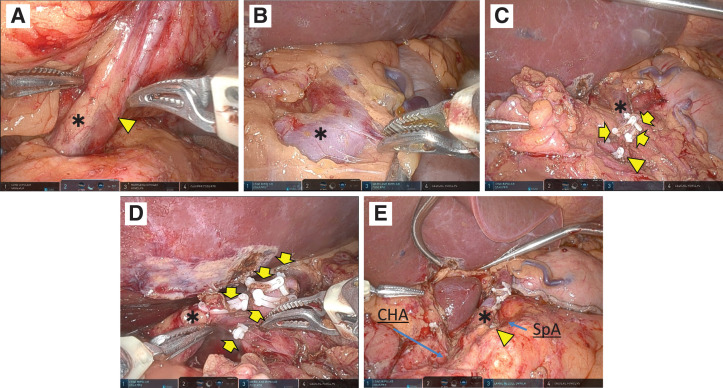
Intraoperative findings during robot-assisted distal gastrectomy. (**A**) Intraoperative identification of the LGA running along the LGV (*), which constituted the CPSS draining into the LRV. (**B**) After clipping and division of the LGA, the proximal dissection line was established. The LGV (*) constituted the main trunk of the CPSS and was preserved throughout the procedure. With the operative view changed to a cranial-to-caudal approach, lymphadenectomy was initiated along the LGV while separating the lymphatic tissue from the vascular wall. (**C**) Drainage veins from the lesser curvature flowing into the shunt vessel were sequentially clipped and divided to facilitate safe lymph node dissection without compromising the shunt vessel (*). The arrows indicate the divided drainage veins entering the shunt vessel. The arrowhead indicates the clipped distal stump of the LGA. (**D**) Using articulated robotic instruments, lymphadenectomy around the CPSS was performed toward the PV side. The preserved LGV (*), which constituted the main trunk of the shunt, was carefully skeletonized while maintaining vascular integrity. The arrows indicate the clipping sites of multiple venous tributaries of comparable diameter draining into the shunt vessel. (**E**) Completion of suprapancreatic lymphadenectomy, confirming that D2 lymph node dissection was appropriately performed en bloc with preservation of the CPSS. The anatomical relationship among the preserved LGV (*), CHA, and SpA is demonstrated after completion of the dissection. The arrowhead indicates the clipped proximal stump of the LGA. CHA, common hepatic artery; CPSS, congenital portosystemic shunt; LGA, left gastric artery; LGV, left gastric vein; LRV, left renal vein; PV, portal vein; SpA, splenic artery

Using articulated robotic instruments, en bloc lymphadenectomy around the shunt vessel was performed toward the PV while maintaining vascular integrity (**Fig. 5C** and **5D**). After proximal gastric transection, it was confirmed that suprapancreatic lymph node dissection had been appropriately completed in accordance with D2 lymphadenectomy, as defined by the Japanese Gastric Cancer Treatment Guidelines (**Fig. 5E**).

Reconstruction was performed using the Billroth II method in a standard manner, and the operation was completed. The operative time was 407 min, and the estimated blood loss was 53 mL.

Postoperative pathological examination revealed an 11 × 11-mm tumor (**Fig. 6**). Immunohistochemical analysis demonstrated positivity for chromogranin A, synaptophysin, and CD56, and the Ki-67 labeling index was <1%, confirming a final diagnosis of a G1 gNET (**Fig. 7A**–**7F**). No lymph node metastasis was observed (0/36). The patient was discharged on POD 7. The postoperative course was uneventful, with liver function tests and serum ammonia levels remaining within the normal range, and no symptoms suggestive of hepatic encephalopathy were observed. Furthermore, contrast-enhanced CT performed 1 month after surgery revealed no evidence of PV thrombosis or other vascular complications.

**Fig. 6:**
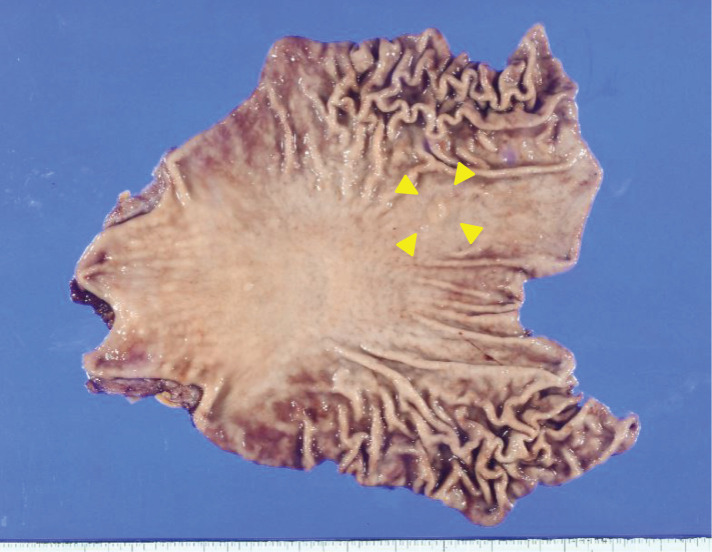
Macroscopic findings of the resected specimen. An 11 × 11-mm elevated lesion is observed on the anterior wall of the gastric mid-body (arrowheads).

**Fig. 7:**
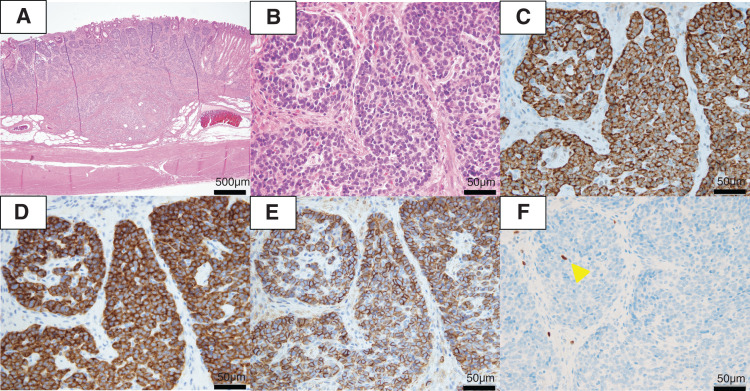
Histopathological and immunohistochemical findings. (**A**) Hematoxylin and eosin staining reveals uniform tumor cells with round nuclei arranged in trabecular and rosette-like patterns (×40). (**B**) Higher magnification shows minimal nuclear atypia and low mitotic activity (×400). (**C**) Immunohistochemical staining demonstrates positivity for chromogranin A (×400). (**D**) The tumor cells are also positive for synaptophysin (×400). (**E**) CD56 staining is diffusely positive (×400). (**F**) The Ki-67 labeling index is <1% (arrowhead) (×400).

## DISCUSSION

gNETs are classified according to the WHO grading system and the Rindi classification; both are crucial for evaluating tumor biology and guiding therapeutic strategies.^3)^ In the present case, analysis of the preoperative endoscopic biopsy revealed a G1 gNET. Although the lesion was confined to the submucosal layer, a Rindi type III gNET was suspected because the serum gastrin level was within normal limits and no evidence of atrophic gastritis was observed. Recent reports suggest that endoscopic or local resection may be considered for selected G1 gNETs measuring ≤10 mm and confined to the submucosal layer. However, in the present case, preoperative endoscopic ultrasonography demonstrated a 13-mm lesion with ulceration, and the possibility of lymph node metastasis could not be completely excluded. Lymph node metastasis occurs in approximately 10%–21% of tumors measuring 1–2 cm in diameter with submucosal invasion.^4,5)^ Therefore, we selected distal gastrectomy with D2 lymphadenectomy adhering to the Japanese Gastric Cancer Treatment Guidelines to ensure oncological radicality.

Portosystemic shunts are classified as cirrhotic or noncirrhotic. Noncirrhotic shunts include congenital anomalies and acquired shunts resulting from trauma or abdominal surgery.^6,7)^ In the present case, the absence of a history of abdominal surgery or trauma suggested a congenital etiology.

A CPSS is a rare vascular anomaly in which portal venous blood drains directly into the systemic circulation, with an estimated incidence of approximately 1 in 30000 births. The most widely used classification system, proposed by Morgan and Superina, divides CPSSs into type I, characterized by the complete absence of intrahepatic portal venous flow, and type II, characterized by hypoplastic intrahepatic portal branches with partial extrahepatic shunting.^8,9)^ In the present case, imaging demonstrated hypoplastic intrahepatic portal branches and a shunt between the LGV and the LRV, leading to a diagnosis of an Abernethy type II malformation.

Adults with a CPSS may present with various clinical manifestations, ranging from an asymptomatic state to hepatic dysfunction, hyperammonemia, and hepatic encephalopathy.^10)^ In the present case, the shunt vessel exhibited radiological features consistent with a CPSS, including a large, straight vessel without collateral circulation or varices.^11,12)^ Shunt closure is generally considered for symptomatic patients with Abernethy type II malformations.^13)^ Although it is widely accepted that symptomatic CPSSs require treatment, the optimal management of asymptomatic adults with a CPSS remains controversial because shunt occlusion may alter portal hemodynamics.^14,15)^ When the shunt fraction (the ratio of shunt flow volume to total portal flow volume) exceeds 60%, the risk of hepatic encephalopathy markedly increases, and intervention is recommended.^16)^ However, quantitative hemodynamic assessments such as shunt fraction analysis, portal pressure measurement, or liver biopsy were not performed in the present case. Therefore, the decision to preserve the shunt was based primarily on the absence of clinical symptoms, the presence of normal liver function and serum ammonia levels, and radiological findings suggesting preserved portal venous flow.

In the present case, preserved portal venous perfusion through the main PV and intrahepatic portal branches may have contributed to the absence of postoperative hepatic dysfunction despite the presence of a CPSS. In addition, the patient exhibited no biochemical or clinical evidence of significant portal flow deprivation, suggesting that hepatopetal portal flow was relatively maintained. Given these findings, we considered preserving the shunt under careful observation to be a reasonable management strategy for this patient.

Given the absence of clinical or laboratory abnormalities, invasive preoperative evaluations were not performed. However, in patients with abnormal laboratory findings or clinical symptoms, liver biopsy and portal pressure measurement may be useful for evaluating hepatic parenchymal damage and portal hemodynamics, provided that the associated risks are carefully considered.^17)^

We conducted a literature review of PubMed and the Japanese medical literature database (Ichushi-Web) covering the period from 1980 to 2026, utilizing the keywords “congenital portosystemic shunt,” “gastric cancer,” “gastrectomy,” and “gastric neuroendocrine tumor.” Only 1 English language report describing gastric cancer concurrent with a CPSS was identified. In the Japanese literature, 4 cases of surgically resected gastric tumors concurrent with a CPSS were identified, including the aforementioned case (**Table 1**).^17–20)^ Among these reports, 3 patients underwent open gastrectomy and 1 underwent laparoscopic surgery; however, we could not identify any report describing robot-assisted gastrectomy preserving a CPSS during gNET resection.

**Table 1:** Reported cases of resected gastric tumors concurrent with a CPSS

No.	Author	Year	Age (years)	Sex	Hyperammonemia	Hepatic encephalopathy	Shunt	Surgical method	Shunt removal
1	Suzumura et al.^18)^	2000	70	M	+	+	LGV–LRV	Distal subtotal gastrectomy	+
2	Sato et al.^19)^	2001	73	M	+	+	Spinal vein–LRV	Distal gastrectomy	+
3	Hamano et al.^20)^	2017	59	M	+	+	LGV–LRV	Laparoscopic distal gastrectomy	+
4	Yanagisawa et al.^17)^	2023	56	M	+	+	LGV–	Distal gastrectomy	+
5	Present case	2026	54	M	–	–	LGV–LRV	Robotic distal gastrectomy	–

CPSS, congenital portosystemic shunt; LGV, left gastric vein; LRV, left renal vein

All previously reported patients exhibited preoperative liver dysfunction or hyperammonemia, and shunt occlusion was performed concomitantly with tumor resection. In contrast, the present case represents a rare instance of a gNET treated by robot-assisted gastrectomy preserving a CPSS in a patient without preoperative hepatic dysfunction.

Based on these considerations, safe gastric resection preserving a CPSS requires detailed preoperative planning. 3D CT was useful for visualizing the vascular anatomy and planning the surgical strategy. We selected robot-assisted distal gastrectomy to facilitate precise dissection while enabling oncological resection. Given the reported 10%–20% incidence of lymph node metastasis in gNETs larger than 1 cm with submucosal invasion, appropriate lymphadenectomy adhering to standard surgical principles is essential.

Robot-assisted surgery provided several technical advantages in the present case. Because strictly avoiding injury to the shunt vessel was essential, the magnified 3D view and articulated robotic instruments facilitated precise lymphadenectomy around the shunt vessel utilizing minimal traction and avoiding applying excessive tension on the anomalous vessel. In addition, small venous tributaries draining into the shunt vessel could be smoothly controlled by coagulation and clipping, which contributed to minimizing intraoperative hemorrhage and ensured the safe completion of the procedure. Consequently, the D2 lymphadenectomy was completed without vascular injury. Furthermore, postoperative pancreatic fistulas can contribute to venous thrombosis in the portal venous system.^21)^ Robotic gastrectomy may reduce pancreas-related complications and intra-abdominal infectious complications compared with conventional laparoscopic gastrectomy.^22)^

In conclusion, this case underscores the importance of individualized surgical decision-making for patients with gNETs concurrent with a CPSS. Comprehensive preoperative evaluation of the vascular anatomy and careful selection of the surgical strategy are essential to ensure oncological radicality and patient safety. When oncologically appropriate, preserving a CPSS should be considered to reduce the risk of postoperative hepatic complications. Therefore, this case suggests that robot-assisted gastrectomy preserving a CPSS may be considered as one possible surgical option for carefully selected patients with gNET and complex vascular anatomy.

Although oncological follow-up for a gNET is mandatory, long-term surveillance is equally important to monitor potential hepatic dysfunction or hyperammonemia secondary to a CPSS. Should such findings develop during follow-up, shunt occlusion may be warranted, given previous reports demonstrating clinical improvement following shunt closure.

This report has several limitations. First, quantitative hemodynamic evaluations, including shunt fraction analysis and portal pressure measurement, were not performed. Therefore, the decision to preserve the shunt was based primarily on clinical, laboratory, and radiological findings rather than direct hemodynamic assessment. Second, the follow-up period remains relatively short, and longer observation is required to evaluate both oncological outcomes and the long-term consequences of preserving the shunt. Consequently, the absence of postoperative complications during this short follow-up period should not be interpreted as definitive evidence of long-term safety.

## CONCLUSIONS

This case demonstrates that gNETs concurrent with a CPSS require individualized surgical strategies based on detailed preoperative assessment of vascular anatomy and hepatic function. In carefully selected asymptomatic patients with a stable CPSS, preserving the shunt represents a viable management option to reduce the risk of postoperative hepatic complications. Robot-assisted gastrectomy combined with preoperative 3D imaging facilitates precise dissection, the safe preservation of anomalous vessels, and oncological lymphadenectomy. Therefore, robot-assisted surgery is a valuable approach in carefully selected patients with complex vascular anomalies, such as a CPSS.

## DECLARATIONS

### Availability of data and materials

Data sharing is not applicable to this article because no datasets were generated or analyzed in this study.
